# Ovarian aging: energy metabolism of oocytes

**DOI:** 10.1186/s13048-024-01427-y

**Published:** 2024-05-31

**Authors:** Shenglan Bao, Tailang Yin, Su Liu

**Affiliations:** 1 Reproductive Medical Center, Renmin Hospital of Wuhan University, Wuhan, China; 2 Shenzhen Key Laboratory of Reproductive Immunology for Peri-Implantation, , Shenzhen Zhongshan Institute for Reproductive Medicine and Genetics, Shenzhen Zhongshan Obstetrics & Gynecology Hospital (Formerly Shenzhen Zhongshan Urology Hospital), Shenzhen, China

**Keywords:** Oocyte, Metabolism, Ovarian aging, TCA cycle, Diminished ovarian reserve, OXPHOS, Lipid metabolism, Glutamine metabolism, Adenosine Remedial Pathway

## Abstract

In women who are getting older, the quantity and quality of their follicles or oocytes and decline. This is characterized by decreased ovarian reserve function (DOR), fewer remaining oocytes, and lower quality oocytes. As more women choose to delay childbirth, the decline in fertility associated with age has become a significant concern for modern women. The decline in oocyte quality is a key indicator of ovarian aging. Many studies suggest that age-related changes in oocyte energy metabolism may impact oocyte quality. Changes in oocyte energy metabolism affect adenosine 5'-triphosphate (ATP) production, but how related products and proteins influence oocyte quality remains largely unknown. This review focuses on oocyte metabolism in age-related ovarian aging and its potential impact on oocyte quality, as well as therapeutic strategies that may partially influence oocyte metabolism. This research aims to enhance our understanding of age-related changes in oocyte energy metabolism, and the identification of biomarkers and treatment methods.

## Introduction

Ovarian aging is a significant cause of female infertility [1]. As a woman ages, the quantity and quality of the follicle or oocyte degenerates, resulting in a decrease in ovarian reserve function (DOR). During this process, fewer oocytes are produced in the ovaries and their quality or ability is diminished. Menopause is the last stage of the ovarian aging process, with most women entering menopause between the ages of 49 and 52. [2]. In modern society, women often postpone childbirth due to a variety of factors, including economics, careers, and lifestyles [3]. However, as humans age, fertility rates begin to decline around 30 years of age and become clinically relevant between the ages of 35 and 40, after which they continue to decline significantly. [4]. The decline in fertility associated with women’s age has become an important issue that troubles modern women.

Changes in the energy metabolism of oocytes due to age can affect the cellular levels of intermediates and byproducts, consequently impacting oocyte quality. However, the mechanisms underlying the effects of changes in the intermediary steps of energy metabolism on adenosine 5'-triphosphate (ATP) generation in oocytes, as well as the influence of related products and proteins on oocyte quality and subsequent ovarian aging, remain unclear.

**Fig.1:**
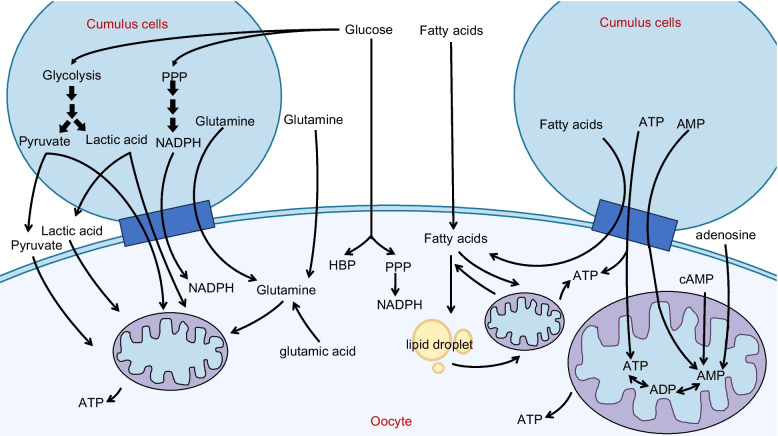
Energy metabolism of oocytes. Oocyte metabolism relies on glucose metabolites provided by cumulus cells. The majority of glucose is metabolized in cumulus cells through anaerobic glycolysis, resulting in lactate production. Cumulus cells can convert glucose into pyruvate, lactate, or nicotinamide adenine dinucleotide phosphate (NADPH) through anaerobic glycolysis and the pentose phosphate pathway. These metabolites are then transferred to oocytes through paracrine signaling and gap junctions, providing energy substrates for oocyte metabolism. Oocytes generate ATP through the tricarboxylic acid (TCA) cycle and oxidative phosphorylation (OXPHOS). Additionally, some glucose can be directly transported to oocytes and metabolized through the pentose phosphate pathway and hexosamine synthesis pathway. Oocytes acquire free fatty acids from the follicular fluid and gap junctions with cumulus cells, and they can also synthesize fatty acids endogenously. After entering the cells, free fatty acids can be converted and stored in lipid droplets or enter mitochondria for β-oxidation. Fatty acids in lipid droplets are esterified and stored as neutral triglycerides (TAGs). Fatty acyl-CoA is synthesized by acyl-CoA synthetases, which catalyze triglycerides into fatty acyl-CoA. Carnitine transports fatty acyl-CoA to mitochondria. The TCA cycle and OXPHOS in mitochondria process fatty acids into acetyl-CoA that is then oxidized, producing ATP once they enter the mitochondrial matrix. Glutamine enters oocytes through the follicular fluid and gap junctions, and oocytes can also synthesize glutamine. Glutamine is metabolized in the mitochondrial matrix as a fuel source for the cycle. Oocytes may possess the ability to convert Adenosine monophosphate (AMP) to ATP through the adenosine salvage pathway. Cumulus cells can also produce ATP through the adenosine salvage pathway and directly supply ATP and AMP to oocytes through gap junctions

The metabolism of energy is important in the development and maturation of oocytes. Energy metabolism processes influence nutrient absorption, macromolecular biosynthesis, energy production, and cellular redox status. The mitochondria-nucleus communication plays a critical role in cellular adaptability, organismal health, and longevity, as well as energy metabolism. [5, 6]. The metabolic pathways of oocytes are complex (Fig. 1). Although cumulus cells produce ATP and provide it to oocytes [7], a decrease in ATP, a decrease in energy production capacity, and a decline in mitochondrial function are all part of the aging of the oocytes [8–15]. Reduced ATP production leads to a decline in oocyte quality, specifically resulting in decreased metabolic activity, which may affect cell cycle regulation, spindle formation during mitosis, chromosome segregation, fertilization, embryo development, and implantation, as discussed in other literature [16–18]. Age-related changes in oocyte energy metabolism can affect the expression of intermediates and byproducts within the cell, thereby influencing oocyte quality. However, it remains unclear how changes in intermediary steps of energy metabolism in oocytes affect ATP generation and how related products and proteins influence oocyte quality, consequently affecting ovarian aging.

Therefore, this review focuses on the oocyte metabolism in age-related ovarian aging and its impact on oocyte quality. This study investigates the relationship between age-related changes in oocyte energy metabolism, decline in oocyte quality, and subsequent decrease in fertility rates. In addition, it helps identify biomarkers and treatment methods.

## Ovarian aging and oocyte energy metabolism

**Fig. 2:**
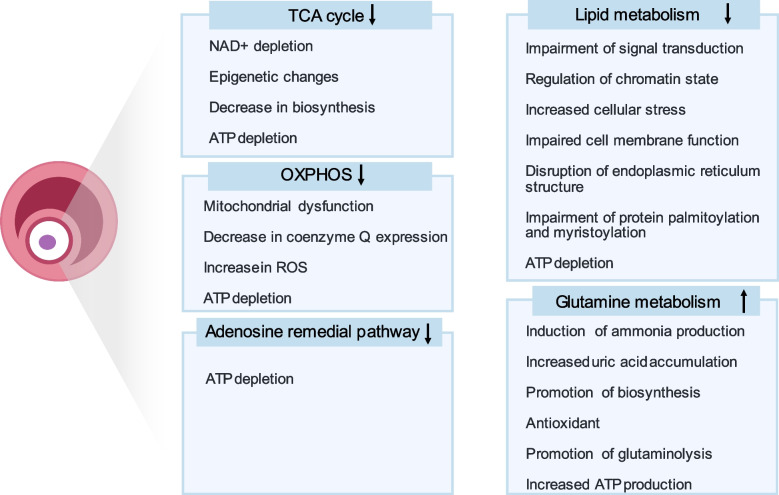
Changes in energy metabolism and potential effects on oocyte

Alterations in several facets of oocyte energy metabolism in individuals suffering from ovarian senescence, such as the Tricarboxylic Acid (TCA) Cycle, Oxidative Phosphorylation, Lipid Metabolism, Glutamine Metabolism, and the Adenosine Remedial Pathway, critically impact the quality of oocytes (Fig. 2).

### TCA cycle

The TCA cycle completely oxidizes the acetyl coenzyme A in cells to produce CO_2_, ATP, nicotinamide adenine dinucleotide (NADH), and flavin adenine dinucleotide (FADH2), and subsequently OXPHOS for the production of ATP. The TCA cycle is a crucial component in signaling pathways and metabolic disorders associated with aging, making it an important target for anti-aging treatment strategies [19, 20]. The TCA cycle takes place in oocytes, and its activity is inhibited with age [7, 11, 21]. Specifically, as age increases, the cross-regional transport of substances such as pyruvate and lactate salts from granulosa cells to oocytes decreases, but metabolites such as pyruvate, lactate salts, and glutamine gradually accumulate in the oocytes [7, 22]. Glucose [7], glucose-6-phosphate [7], sorbitol [13], mannitol [13], urea cycle intermediates such as aspartate [7], ornithine [7], and arginine [7] increase in oocytes of older mothers, indicating that energy substrates are diverted to the pentose phosphate pathway, hexosamine synthesis pathway, and urea cycle; the TCA cycle cannot process available substrates. Furthermore, the TCA cycle intermediates succinate [7], fumarate [7], citrate [11], isocitrate [11], and malate [11] decrease in an age-dependent manner in oocytes. The reduced levels of NAD + and FAD in oocytes are also observed [7, 11, 23–26]. The age-related changes in TCA cycle activity differ between species. For example, in the oocytes of horses, although the glucose abundance in the cumulus cells of older horses is higher, the level of pyruvate in the oocytes of older mares is consistently lower than that of young mares during the GV, MI, and MII stages. This suggests impaired transport or production of pyruvate, possibly due to reduced transzonal transport [13, 22]. These differences may be attributed to variations in samples and species.

The activity of TCA cycle metabolism decreases with age, leading to reduced levels of NAD + restoration [7, 23–25]. NAD + plays a central role in controlling hundreds of pathways in both energy metabolism and cell survival. Both NAD + and its reduced form are involved in various biological processes [20, 27–30]. Increased NAD + production or decreased degradation appears to be profitable, as reduced NAD + levels can lead to metabolic and age-related diseases [31]. In terms of aging, NAD + is essential in antioxidation, mitochondrial function, central carbon metabolism, cellular aging, protein deacetylation, and DNA damage [20, 29, 30]. Some enzymes consume NAD + , such as the sirtuin enzyme family (SIRTs) and poly(ADP-ribose) polymerase (PARP) [20]. These enzymes have become critical factors in aging [20]. In mouse ovaries with a knockout of NAD + synthesis genes, NAD + levels decrease in mid-aged mice, resulting in the impairment of oocyte quality, characterized by increased abnormal spindle and reactive oxygen species (ROS) formation [32]. Supplementation of NAD + precursor nicotinamide riboside (NR) can increase ovarian reserve and improve oocyte quality [32, 33].

The alterations in the TCA cycle intermediates can impact oocyte quality. The levels of TCA cycle intermediates, including succinic acid [7], jasmonic acid [7], citrate [11], malate [11], and fumarate [11], decrease with age in oocytes and can influence epigenetic changes. Decreased levels of succinic acid and fumarate can influence the levels of DNA and histone methylation, while decreased levels of citrate can weaken its ability to enhance histone acetylation. These effects can further contribute to the aging process [34, 35]. In addition, the TCA cycle is also related to metabolite production and biosynthesis. Intermediates of the TCA cycle can serve as precursors for amino acid synthesis, nucleotide synthesis, and fatty acid and cholesterol synthesis. In oocytes, oral administration of dimethyl fumarate can alleviate oxidative stress and delay age-related infertility in mice ovaries [36]. Moreover, the decrease in citrate levels within the follicular fluid has the potential to impact the process of oocyte maturation [37].

### Phosphorylation of oxidation

The decline in oxidative phosphorylation caused by mitochondrial dysfunction is an important marker of human aging [38]. In oocytes, the energy released from glycolysis and the TCA cycle is mostly stored in reduced coenzymes and needs to be synthesized into ATP through the oxidative phosphorylation process in the mitochondria [21]. The respiratory chain consists of more than 15 components, mainly including NADH dehydrogenase (complex I), succinate dehydrogenase (complex II), cytochrome c oxidoreductase (complex III), cytochrome c oxidase (complex IV), coenzyme Q (CoQ), and cytochrome C. The electron transport chain (ETC) facilitates the translocation of protons (H +) from the matrix to the intermembrane space, consequently establishing a proton-motive force (PMF). The energy produced by PMF is used by ATP synthase to phosphorylate adenosine diphosphate (ADP) into ATP. There are two non-exclusive mechanisms for regulating oxidative phosphorylation. It can be dynamically regulated, enabling an adjustment in ATP synthesis rate to meet ATP demand [10]. Oxidative phosphorylation can also be regulated by altering the number of mitochondria [10]. OXPHOS is highly active in oocytes [39]. With age, ATP generation through oxidative phosphorylation in oocytes decreases, and the function of ETC is impaired [12–14]. The expression of the majority of genes encoding subunits of respiratory chain complexes I to V is downregulated [11, 23, 40]. In addition, the expression of genes involved in the synthesis of CoQ [11, 23] and the protein ADP/ATP translocase 1 (ANT1) that mediates mitochondrial ATP/ADP transport decreases in an age-dependent manner in oocytes [11, 23, 41]. Studies have also shown that the expression of OXPHOS is upregulated in mouse and human oocytes with age in the field of translational genomics, but the translational efficiency of the OXPHOS pathway in aged mouse oocytes is lower than in young mouse oocytes [15]. The expression of mitochondrial DNA (mtDNA) encoding subunits involved in the respiratory chain also decreases in an age-dependent manner in oocytes [40]. Mitochondrial ribosomes are ribosomes present in the mitochondria of eukaryotic cells. These genes encoding mitochondrial ribosomal proteins are downregulated in oocytes of aged rhesus monkeys [23]. In addition, mutations in mtDNA increase in an age-dependent manner in oocytes [42].

In addition to energy metabolism, the components of the respiratory chain have other important roles in cells. CoQ is downregulated in oocytes [43–46]. As the primary liposoluble antioxidant, CoQ also exerts anti-inflammatory and anti-apoptotic effects through gene expression regulation [43–46]. Insufficient CoQ production in oocytes can lead to defects in mitochondrial performance and a decline in reproductive performance, and CoQ10 treatment can reverse changes in mitochondrial function in aging oocytes [11, 47]. Age-dependent decline in respiratory chain complexes I and III in oocytes are deemed as sources of mitochondrial ROS [48, 49]. Complex I is a vital source of ROS in the reverse electron transfer process of complex II [50]. Cytochrome c can also regulate the efficiency of OXPHOS and reduce the production of ROS [51]. In a state of normal physiology, ROS levels are controlled by the antioxidant enzyme system. However, when the ROS levels produced by damaged OXPHOS exceed the defense capacity, it can have adverse effects on cell quality [52–54]. Previous studies have shown that oxidative stress increases with age in oocytes [7]. Impaired respiratory chains in oocytes [11, 23, 40, 51, 55] may lead to excessive production of mitochondrial ROS beyond the defense capacity, resulting in adverse effects on oocyte quality [48–51, 53, 54, 56]. It is important to consider that the downregulation of mitochondrial respiratory chain expression can also promote healthy aging. Studies have demonstrated that partial inhibition of ETC can prolong the lifespan of nematodes [57, 58]. Disturbance of OXPHOS, impaired complex assembly, excessive ROS, and accumulation of misfolded proteins can induce beneficial mitochondrial integrated stress response and promote healthy aging [59, 60]. This has been studied in age-related neurodegenerative diseases [60]. However, the enhanced mitochondrial stress response in oocytes may lead to female infertility, impaired oocyte maturation and blastocyst development, failure to form embryos, accelerated follicular depletion, and a phenotype resembling premature reproductive aging [61]. It is worth noting that in cardiomyocytes, aging can be delayed by inhibiting proton leakage through proteins such as ANT1 [62, 63]. However, the specific mechanism is not yet clear, and the relationship between the downregulation of ANT1 expression and oocyte quality is not fully understood. In addition, inhibition of ATP synthase can extend the lifespan of nematodes [64]. However, the role of decreased ATP synthase activity in age-dependent decline in oocyte quality is not well understood. Furthermore, age-dependent decrease in mtDNA expression and increased mutations in oocytes may affect mitochondrial function, and susceptibility to oxidative damage, and lead to a decline in fertility with age, as well as harmful effects on offspring [65]. Supplementing mtDNA copy numbers can improve oocyte quality [66–68].

### Lipid metabolism

**Fig. 3:**
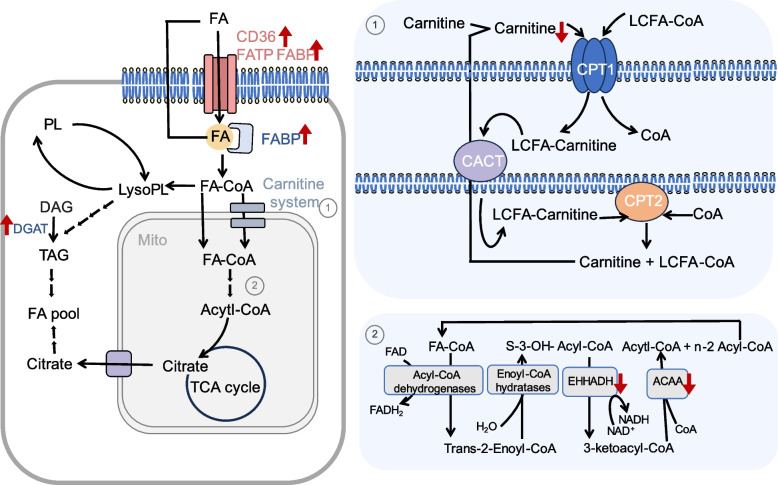
Changes in oocyte lipid metabolism-related substances. Oocytes acquire free fatty acids from follicular fluid and the interstitial space connected to cumulus cells, and they can also synthesize fatty acids internally. Fatty acids (FAs) are transported into oocytes through fatty acid translocase (CD36), fatty acid transport protein (FATP), or other mechanisms. They are carried by fatty acid-binding protein (FABP) and converted into fatty acyl-CoA. CD36 and FABP3 are upregulated in oocytes in an age-related manner. Fatty acyl-CoA is used for the synthesis of structural lipids or lipid storage, or it can enter the tricarboxylic acid (TCA) cycle as acetyl coenzyme A (Acetyl-CoA) through the fatty acid β-oxidation (FAO) pathway. The carnitine system facilitates the transport of fatty acyl-CoA into mitochondria, which involves the participation of carnitine. Carnitine shows an age-related decline in oocytes. 3-ketoacyl-CoA thiolase (ACAA) and enoyl-CoA hydratase 3-hydroxyacyl-CoA dehydrogenase (EHHADH) are important proteins involved in FAO, and they also exhibit an age-related decline in oocytes. Additionally, diacylglycerol o-acyltransferase 1 (DGAT1) is an important protein for cellular fatty acid synthesis, and it is upregulated in oocytes in an age-related manner

A decline in the fatty acid oxidation capacity of oocytes may lead to insufficient energy utilization, especially when oocytes rely on fatty acid oxidation [69–73]. As age increases, lipid metabolism changes occur in oocytes (Fig. 3). The fatty acid oxidation capacity of oocytes decreases, which may serve as the cause of the accumulation of free fatty acids and a decrease in the abundance of long-chain polyunsaturated fatty acids. Studies have shown that some important proteins in lipid metabolism, such as diacylglycerol o-acyltransferase 1 (DGAT1) [23], cluster of differentiation 36 (CD36) [23, 74], and fatty acid-binding protein 3 (FABP3) [23] are upregulated in an age-dependent manner in oocytes, indicating an enhanced ability of oocytes to absorb, transport, and synthesize triglycerides. However, the activities of fatty acids metabolism-related substances such as carnitine [7], 3-ketoacyl-CoA thiolase (ACAA) [75], and enoyl-CoA hydratase 3-hydroxy acyl-CoA dehydrogenase (EHHADH) [75] in oocytes show an age-dependent decline. N-acyl ethanolamine, as a signaling molecule, promotes fatty acid breakdown and inhibits fatty acid synthesis, but its expression is downregulated in oocytes of aged mares [13]. These factors lead to lipid accumulation and increased abundance of free fatty acids in oocytes, while inhibiting fatty acid oxidation, impacting oocyte quality in multiple ways. Specifically, DGAT catalyzes the final step of triglyceride biosynthesis, which is the rate-limiting enzyme. DGAT1 inhibition reduces lipid content, increases mitochondrial activity, and promotes lipid metabolism regulation and oxidative stress-related transcription. [76]. In addition to facilitating the uptake and transportation of long-chain fatty acids, CD36 can also enhance oxidative function by upregulating the expression of peroxisome proliferator-activated receptor alpha (PPARα), thereby reducing lipid deposition [77–81]. The function of CD36 is related to its subcellular localization [82, 83]. However, the changes in CD36 subcellular localization in aging oocytes have not been fully studied. FABP can transport fatty acids within cells and has characteristics of intracellular and extracellular FA transport similar to CD36 [84]. Increased expression of FABP3 can lead to lipid accumulation in oocytes [84]. Carnitine, a molecule promoting the entry of fatty acyl-coenzyme A into mitochondria, is crucial for fatty acid oxidation. It is worth noting that although carnitine expression overall decreases, the age-dependent expression of butyryl carnitine and acetylcarnitine increases in oocytes [7]. Carnitine treatment contributes to the preservation of oocyte quality and the enhancement of embryo development [85–89]. Specifically, it decreases oocyte lipid content, boosts cellular energy supply, enhances antioxidant capacity, and actively modulates mitochondrial activity during oxidative stress [85–89]. This, to some extent, prevents abnormal changes in phospholipid and sphingolipid content as oocytes develop into blastocysts [85–89]. However, the specific changes in acylcarnitine spectrum in oocytes with age and its potential clinical value have not been well explored. ACAA and EHHADH are essential proteins in the fatty acid β-oxidation (FAO) process, and their downregulation is associated with decreased efficiency. FAO is critical for oocyte nuclei maturation [90, 91]. Stimulation of FAO can decrease lipid storage, positively impacting subsequent embryo development [85, 90–92]. Excessive fatty acids or reduced FAO can negatively impact oocyte quality, leading to cellular stress responses and affecting pre-implantation embryo quality [85, 90–92].

Additionally, fatty acids are important raw materials for the synthesis of phospholipids. Supplementing fatty acids can alleviate the abnormal changes in phospholipid and sphingolipid content when culturing oocytes in vitro to develop into blastocysts fatty [88].but the age-dependent expression of phospholipids in oocytes decreases [7, 13], indicating a decrease in the utilization level of fatty acids by oocytes. Consistently, the abundance of triglycerides in the oocytes of aged mares is higher than that of young mares [13], and the total amount of free fatty acids in the oocytes of young horses is higher than that in the oocytes of old horses [12]. However, some studies have shown that the total abundance of triglycerides in the oocytes of mares is similar between young and old age groups [12], which may be due to sample differences. Additionally, the abundance of long-chain polyunsaturated fatty acids is lower in oocytes of aged mares [13]. In GV oocytes, there is no significant difference in lipid composition between young and old horses [13].

The decline in fatty acid oxidation capacity in oocytes with age, the accumulation of free fatty acids in the cytoplasm, and the decrease in abundance of long-chain polyunsaturated fatty acids are all associated with oocyte quality. These factors can impact signal transduction, chromatin regulation, cellular stress levels, cell membrane function, endoplasmic reticulum structure, as well as protein palmitoylation and myristoylation [7, 12, 13, 23, 73, 75, 93–97]. Additionally, unsaturated fatty acids reduce saturated fatty acid toxicity [98, 99]. Polyunsaturated fatty acids (PUFAs) are crucial structural components of mammalian membrane systems and signaling molecules, encompassing essential fatty acids like linoleic acid and α-linolenic acid, as well as long-chain and very long-chain ω3 and ω6 polyunsaturated fatty acids. Common PUFAs comprise linoleic acid, oleic acid, arachidonic acid, and docosahexaenoic acid (DHA). Inadequate PUFA synthesis may result in reduced oocyte quality, arrested follicle development, oocyte meiotic arrest, and infertility [100, 101]. Long-term consumption of a high-PUFA diet or short-term dietary therapy initiated when age-related rapid decline in reproductive function begins in mice can extend reproductive function [102].It is important to highlight that different types of PUFAs affect oocyte quality differently. ω3-PUFAs can decrease abnormal oxidative stress levels and spindle/chromosome aberration rates in oocytes [103]. The consumption of long-chain ω3-PUFAs safeguards reproductive function and is linked to a greater likelihood of successful reproduction in women undergoing assisted reproduction [104, 105]. However, research has indicated that exposing oocytes to environments rich in ω3-PUFAs may adversely affect oocyte quality, leading to changes in mitochondrial distribution, calcium levels, and increased production of reactive oxygen species during ovarian maturation [106]. Additionally, ω3-PUFAs-rich environments can impair oocyte morphology, reduce oocyte proliferation, and inhibit blastocyst formation [106]. In the follicular fluid of large follicles, ω3-PUFAs show a positive correlation with female age and a negative correlation with total oocyte number, mid-cycle oocyte number, total embryo number, high-quality embryo number, and fertilization rate [100, 107]. However, this study cannot exclude the possibility that the increase in these ω-3 fatty acids in follicular fluid is due to decreased oocyte absorption rates. DHA is an ω3-PUFA that plays a crucial role in oocyte quality. Low-dose DHA can enhance oocyte developmental ability in vitro, leading to significant improvements in cleavage rate, embryo rate after parthenogenetic activation, and blastocyst rate on the 7th day after in vitro fertilization [108]. Endogenously synthesized DHA in oocytes can enhance the reproductive capacity, maturation, and quality of oocytes in zebrafish [101]. DHA deficiency can result in oocyte activation defects, poor microtubule stability, and reduced pregnenolone levels, which may be partially attributed to DHA’s regulation of pregnenolone production [101].But in the follicular fluid of large follicles, DHA is positively correlated with female age but negatively correlated with the total number of oocytes, mid-stage oocytes, total embryos, high-quality embryos, and fertilization rate [107]. This study cannot exclude the possibility that the increase in DHA in the follicular fluid results from decreased oocyte absorption [107]. Unlike ω3-PUFA, the correlation between ω6-PUFA content and oocyte quality appears to be low. A diet rich in ω6-PUFA is not significantly associated with the reproductive success of aged female mice [102]. The serum ω6-PUFA, ω6/ω3-PUFA ratio, and total PUFA of women undergoing assisted reproduction are not related to the outcomes of assisted reproduction [105]. Unlike ω3-PUFA, several ω6-PUFA and ω6/ω3-PUFA ratios in the follicular fluid of large follicles decrease with age. The roles of oleic acid, linoleic acid, and arachidonic acid in oocyte development are not fully understood. Research has demonstrated that supplementing oleic acid during in vitro cultivation enhances the blastocyst formation rate. [109]. Linoleic acid may regulate oocyte development, but high concentrations are linked to decreased oocyte quality, impairing maturation, in vitro maturation, and embryo development [110–112]. Arachidonic acid, a precursor of prostaglandins, is involved in reproductive processes including follicle development, oocyte maturation, ovulation, and embryo implantation. Studies have indicated that regulating arachidonic acid metabolism can help restore fertility in patients with premature ovarian failure [113]. However, high concentrations of arachidonic acid are associated with decreased oocyte quality [110]. In summary, while high concentrations of PUFA in follicular fluid and oocytes may have adverse effects, different categories of PUFA have varying impacts on oocyte quality. Excessive lipid accumulation in oocytes can lead to decreased oocyte quality. The high levels of lipids in oocytes can impair oocyte developmental capacity and reduce cold storage survival rates [114–117]. This can also lead to abnormalities in cell energy metabolism, cell death, and inflammation pathways [114–117]. Whether in vivo or in vitro, exposure of oocytes to a lipid-rich environment is detrimental to oocyte development, resulting in delayed oocyte maturation, low fertilization rates, reduced blastocyst formation, fewer blastocyst cells, high apoptosis rates, and metabolic abnormalities [118–124]. These factors are also significant contributors to infertility in obese women [118–124]. Excessive lipid accumulation's exact mechanisms on oocyte quality remain incompletely understood. However, it likely involves triggering cascade reactions of lipid toxicity within oocytes, disrupting endoplasmic reticulum structure, and affecting various pathways, collectively resulting in decreased oocyte quality. [96, 125, 126].

### Glutamine metabolism

Glutamine is the most abundant and widely used amino acid in the body [127, 128]. It is also an essential source of carbon in oocytes [72, 129–131]. Several enzymes are involved in glutamine-related energy metabolism, with glutamine synthetase (GS) and phosphate-dependent glutaminase (GLS) being the two major intracellular enzymes [130]. GS catalyzes the conversion of glutamine from glutamate, while GLS catalyzes the conversion of glutamine to glutamate. Glutamate is then transformed into alpha-ketoglutarate through the action of mitochondrial GDH, entering the TCA cycle [130, 132]. Therefore, glutamate is an important intermediate substance for glutamine entering energy metabolism. In oocytes, glutamine accumulates with age. The abundance of glutamine in the GV and MI stage oocytes of elderly women is higher than that of young women [7]. Compared with young mares, glutamine levels are higher in oocytes and follicular cells of old mares [13]. In addition, omics analyses of human oocytes and cumulus cells from advanced maternal age have shown that glutamine accumulates with age [26]. However, the age-dependent decline in TCA cycle activity in oocytes and the reduced metabolic capacity for utilizing glutamine suggest that the increase in glutamine abundance may have limited effects on enhancing cellular energy metabolism. The ability to metabolize glutamine varies among species. In secondary and antral follicles of aged crab-eating monkeys, GLS2 expression increases in oocytes [23], indicating an age-dependent enhancement of glutamine degradation ability in monkey oocytes.

Apart from energy metabolism, an increased abundance of glutamine in oocytes may affect aging through other pathways. For instance, on one hand, the increased abundance of glutamine in aging cells and enhanced glutamine decomposition can induce ammonia production, which can neutralize the decreased pH in aging cells due to lysosomal membrane damage, thus protecting cell survival [133]. On the other hand, an increased abundance of glutamine and increased activity of the key enzyme GLS1 in aging cells can lead to uric acid accumulation, resulting in mitochondrial dysfunction and DNA damage in cells [134]. However, age-dependent changes in uric acid expression in oocytes have not been reported. Additionally, the urea transporter solute carrier family 14 member 1 (SLC14A1) can transport urea out of cells and its age-dependent expression is increased in oocytes of aged crab-eating macaque primordial follicles [23]. Therefore, further research is needed to investigate whether the increased abundance of glutamine can affect oocyte quality through urea accumulation. Glutamine can serve as a nitrogen donor (α- and γ-nitrogen) or a carbon donor, promoting the biosynthesis of important products such as NAD + [127, 128]. Glutamine can exert antioxidant effects by maintaining intracellular levels of glutathione [135]. Glutamine can also undergo glutathionylation, a process of glutamine-protein modification, which is highly conserved in all metazoans and protists and plays a vital role in many physiological and pathological processes [136]. However, further research is needed to understand how the increased abundance of glutamine affects oocyte aging by influencing intracellular biosynthesis.

### Adenosine remedial pathway

The age-dependent decline in adenosine recycling pathway activity of oocytes. Although the expression of phosphodiesterase (PDE) subtypes PDE8A, PDE3A, PDE8B, and PDE4A in oocytes of aged crab-eating macaques increased [23], indicating an age-dependent increase in the ability of oocytes to break down cAMP into AMP. However, the AMP levels decreased in GV oocytes and MI oocytes of elderly pregnant women, and the levels of phosphocreatine and creatine in MI oocytes decreased [7]. The adenosine content in MII oocytes of mares was lower than in young mares [13]. The age-dependent downregulation of ATP production in oocytes was observed [8, 9], indicating a decline in the activity of the adenosine recycling pathway in oocytes. However, changes in cAMP and ADP levels in oocytes with age have not been reported.

### Glycolysis and pentose phosphate pathways

Glucose is the primary carbon source for most mammalian cells and is essential for oocyte maturation [137–139]. Studies have demonstrated that oocyte glucose utilization depends on the presence of cumulus cells; in the absence of cumulus cells, oocytes struggle to utilize glucose [140, 141]. Within the follicle, cumulus cells metabolize the majority of glucose and can convert it into pyruvate, lactate, or NADPH primarily through anaerobic glycolysis and the pentose phosphate pathway (PPP) [39, 140–150]. They then transfer these products to the oocytes through paracrine and gap junctional communication [39, 140–150]. Notably, research has indicated that the pentose phosphate pathway, rather than glycolysis, is crucial for oocyte maturation [138, 139, 148, 150, 151]. The specific mechanism by which the PPP plays a necessary role is still unclear. In addition to the influence of the PPP product NADPH, this may be partly due to potential defects in glycolysis in cumulus cells, and the generation of pyruvate may depend on intermediate processes of the PPP [138–140, 148, 151, 152]. In contrast to cumulus cells, oocytes have lower glucose metabolism capabilities. They do not exhibit insulin-stimulated glucose uptake and have lower glycolytic activity, relying more on obtaining pyruvate, lactate, or NADPH directly from cumulus cells and follicular fluid [39, 140–143, 145–147, 153, 154]. Glucose metabolism products play a crucial role in oocyte maturation. Pyruvate maintains energy supply and intracellular redox potential for oocytes, promotes the maturation of oocyte nucleus and cytoplasm, inhibits oocyte aging, and affects oocyte transcriptional activity and lipid metabolism through the formation of acetyl-CoA [39, 139–150, 155, 156]. Lactate also affects oocyte quality through the formation of pyruvate [39, 140–150, 155, 156]. Additionally, NADPH controls cellular oxidative stress and promotes the biosynthesis of substances such as tetrahydrofolate, deoxyribonucleotides, proline, fatty acids, and cholesterol [152]. Metabolomic analysis has shown that glucose, pyruvate, and lactate accumulate in human oocytes with age [26]. NADPH expression decreased in the oocytes of aged mice [157]. The activation status of pyruvate dehydrogenase (PDH), a key protein that inputs pyruvate into the TCA cycle, is unrelated to the age of human oocytes [26]. Therefore, it possibly due to a decline in the TCA cycle capacity and the processing capacity of raw materials in oocytes.

## Regulatory mechanism

### Key proteins regulating energy metabolism in aging oocytes

Sirtuins (SIRTs 1–7) are protein deacetylases/ADP-ribosyltransferases involved in various biological functions. They consist of seven members and are expressed in oocytes [158]. Sirtuins play important roles in energy metabolism in oocytes. During reproductive aging, the expressions of SIRT1-3 decrease in oocytes [159–164]. However, the expressions of SIRT4 and SIRT6 increase [23, 165]. There is also evidence suggesting an age-dependent increase in SIRT1 expression in oocytes [23]. SIRT3 and SIRT4 are key regulatory factors in mitochondrial energy metabolism, preventing aging and age-related metabolic abnormalities. For example, SIRT3 promotes ATP production in the ETC, enhances the TCA cycle, promotes glutamine metabolism, and inhibits FAO [166–172]. In oocytes, the lack of SIRT3 leads to the accumulation of superoxide and mitochondrial dysfunction, as well as increased expression of aging-related genes [162, 173]. Although SIRT4 expression increases ATP concentration by reducing respiratory chain uncoupling [174], it can also post-translationally inactivate mitochondrial GDH, reduce PDH activity, and inhibit mitochondrial trifunctional protein α-subunit (MTPα), thereby decreasing TCA cycle activity and FAO [175–178]. Overexpression of SIRT4 results in incomplete meiosis, insufficient mitochondrial redistribution, decreased ATP levels, increased ROS levels, severe spindle/chromosome disorganization, and partial rescue of age-related defects in oocytes through SIRT4 knockout [165, 173]. SIRT6 is a core regulatory factor in energy metabolism, partially achieved through the regulation of SIRT3 and SIRT4 [179]. The deficiency of SIRT6 leads to reduced mitochondrial gene expression, changes in TCA cycle by-product metabolism, downregulation of NAD + , NADH, and ADP, increased ROS production, decreased mitochondrial quantity, and impaired membrane potential [179]. In addition, similar trends were observed in other metabolites associated with energy and carbohydrate metabolism pathways in SIRT6-deficient cells, with only four metabolites upregulated and the remaining 14 downregulated, as well as significant changes in metabolomic features constituting lipid and amino acid metabolism pathways [179]. In oocytes, the meiotic defects caused by SIRT6 inhibition may be due to early apoptosis induced by excessive ROS [180]. Apart from SIRT3 and SIRT4, other Sirtuins also influence cellular metabolism. SIRT2 regulates the intercellular connection between oocytes and cumulus cells, so a decrease in SIRT2 expression may affect nutrient acquisition by oocytes [13, 181]. SIRT2 deacetylates electron transport chain complexes, thereby promoting ATP generation [167]. It can also affect mitochondrial function by influencing the cellular redox state in oocytes [163, 182]. SIRT1 regulates mitochondrial activity and is one of the protective factors against ovarian oxidative stress and glucose stress [162, 173, 183–185]. Enhancing SIRT1 can protect oocyte quality and delay reproductive aging [186]. Inhibiting SIRT1 impairs mitochondrial function and OXPHOS [187]. It can also stimulate follicular apoptosis [187].

Forkhead box O3a (FoxO3a) is a member of the forkhead box O (FoxO) protein family, which is involved in cell survival and lifespan regulation and can influence cellular energy metabolism [188–190]. The expression of FoxO3a is negatively correlated with aerobic glycolysis, fatty acid oxidation, and ATP generation. It can decrease the expression of pyruvate kinase isozymes and lactate dehydrogenase, thereby reducing the metabolites entering the TCA cycle and lowering the level of respiratory chain complexes [190–194]. The downregulation of FoxO3a expression promotes mitochondrial membrane potential hyperpolarization, oxygen consumption, and accumulation of lipid peroxides [190]. Moreover, FoxO3a is present in mitochondria and interacts with SIRT3, which constitutes a potential mitochondrial signaling cascade pathway [195]. With age, the expression of FoxO3a increases in aging oocytes and its cellular localization shifts from the cytoplasm to the chromatin [196]. Increased expression of FoxO3a can affect mitochondrial function in aging oocytes [196, 197]. Inhibiting FoxO3 can protect primordial follicles from excessive activation [198]. However, there are also studies suggesting that FoxO3 can maintain ovarian reserve in aged mice [199]. FoxO3a can be regulated by the PI3K/PTEN/AKT pathway, as well as the mammalian target of rapamycin (mTOR) and adenosine monophosphate-activated protein kinase (AMPK) [188–190, 200]. These pathways may affect metabolism by modulating the levels of FoxO3a. Additionally, the expression of FoxO3a is related to the growth hormone (GH) and insulin-like growth factor (IGF). Increased GH is related to decreased ovarian primordial follicle reserve and an increase in the phosphorylated FoxO3a content in oocytes [201]. In muscle, Insulin and IGF-1 receptors regulate mitochondrial bioenergetics and supercomplex formation through FoxOs dependent on complex I [202]. However, the regulation of oocyte metabolism by GH and IGFs has not been thoroughly investigated.

### Epigenetic inheritance of energy metabolism in aging oocytes

Epigenetics involves heritable changes in chromosomes that do not affect the DNA sequence, including DNA methylation, histone modification, and non-coding RNA, which are important for oocyte development. [203–206]. Additionally, RNA epitranscriptomes significantly impact oocyte quality [207–213]. The epigenome of oocytes changes with age, potentially contributing to the decline in oocyte quality associated with aging [206, 214–218]. Epigenetics can also reshape cellular metabolism by participating in metabolic reprogramming through the regulation of metabolic enzymes and upstream regulator expression and function. Age-related changes in the oocyte epigenome may influence the energy metabolism process of oocytes during ovarian aging, thereby affecting oocyte quality.

DNA methylation involves the enzymatic addition of a methyl group by DNA methyltransferases (DNMT) to the 5’ carbon of cytosine within a cytosine-phosphate-guanine (CpG) dinucleotide context, resulting in the formation of 5-methylcytosine (5-mC). [219, 220]. This process silences gene expression by stabilizing chromatin structure [219, 220]. Furthermore, DNA methylation can recruit chromatin modifiers through methyl-CpG binding domain proteins, which recognize 5-mC and modify histones, affecting chromatin compaction to enhance gene silencing [221]. Additionally, DNA methylation includes specific cases such as ribosomal DNA (rDNA) methylation and mitochondrial DNA methylation, which are related to the transcription activity of ribosomal and mitochondrial proteins. The overall DNA methylation patterns of young and old female oocytes are similar, with characteristic DNA methylation landscapes of alternating high-methylation and low-methylation domains preserved in oocytes [218]. However, total CpG methylation is slightly reduced, and non-CpG methylation is increased in the older group [218]. rDNA methylation increases with age in oocytes [222]. Mitochondrial DNA methylation is absent in mature mouse oocytes, early embryo development, and aging after oocyte ovulation, which may lead to increased protein transcription of mtDNA [223]. However, the specific role and mechanism require further study [16]. Additionally, it is worth noting that the degree of methylation in oocytes of the same individual may vary greatly [222]. Changes in oocyte DNA methylation with age may impact oocyte energy metabolism. For example, the knockdown of Homeobox D8 (HOXD8) can enhance the expression of Phosphoinositide 3-kinase (PI3K) and phosphorylated Ak Strain Transforming (p-AKT) proteins in breast cancer cells [224]. PI3K and p-AKT are crucial proteins that control energy metabolism and impact cellular metabolism by directly regulating nutrient transporters and metabolic enzymes, or by influencing transcription factors that affect metabolic pathways [225]. In oocytes, the CpGs of HOXD8 are positively correlated with age [226]. This may lead to reduced HOXD8 transcription, affecting the expression of PI3K and p-AKT, thus affecting energy metabolism, but further research is needed to confirm this. mTOR is a well-known molecule that regulates metabolism [227]. Mitogen-activated protein Kinase Associated Protein 1 (MAPKAP1) is the main partner of mTORC2, acting as a scaffold and responsible for the substrate specificity of mTOR catalytic subunits [228]. Therefore, the CpGs of MAPKAP1 in oocytes are negatively correlated with age, possibly affecting oocyte energy metabolism by upregulating mTOR activity, but this also needs experimental verification [226]. Knockout of transcription co-activator factor 20 (Tcf20) in embryonic liver and mouse embryonic fibroblasts results in differential expression of proteins related to the mitochondrial oxidative phosphorylation chain, increased mitochondrial metabolic activity, and alterations in TCA cycle metabolites [229]. In oocytes, the CpGs of Tcf20 show a negative correlation with age [226]. This may enhance the transcription of Tcf20, thereby influencing mitochondrial oxidative phosphorylation and TCA cycle activity. However, this relationship has not yet been investigated in oocytes. Hence, DNA methylation could significantly impact the energy metabolism of aging oocytes, but relevant research remains incomplete.

Histone modification encompasses the chemical alterations of amino acids on histones, primarily through processes such as methylation, acetylation, and phosphorylation [230]. These modifications undergo significant age-related changes in oocytes, influencing energy metabolism. Specifically, aging correlates with variations in histone methylation levels and a marked decrease in the expression of histone methyltransferases like lysine acetyltransferase 8 (KAT8), Histone Deacetylase (HDAC), and SIRTs [206, 216, 231–235]. Notably, methylation at H3K4 decreases with age in oocytes [206, 216, 231–234]. The aberrant histone modification, H3.3-K4M, leads to substantial reductions in mtDNA copy number, membrane potential, and ATP content, and escalates ROS levels, along with altering mitochondrial dynamics regulators, oxidative stress-related protein expression, and hydrogen peroxide accumulation [231]. Consequently, changes in H3K4 methylation associated with aging in oocytes may adversely affect mitochondrial dynamics and promote oxidative stress. In contrast, histone acetylation levels in oocytes remain relatively unchanged despite a decrease in HDAC expression [206, 234, 236, 237]. Nonetheless, specific histone deacetylation markers, such as H4K12ac and H4K16ac, exhibit age-related declines [234, 236]. Research on histone phosphorylation in aging oocytes is limited, and the roles of histone acetylation and phosphorylation in the metabolic processes of aging oocytes are not yet fully understood, with only sporadic studies reported. Overall, it remains unclear which genomic sites in oocytes are susceptible to age-related histone modifications and their impact on energy metabolism.

Non-coding RNAs (ncRNAs) are a unique class of RNA molecules that play a crucial role in regulating energy metabolism and have diverse functions in the ovary [238]. Differentially regulated ncRNAs in oocytes may impact energy metabolism. For instance, Nuclear receptor subfamily 1, group D, member 1 (NR1D1) is crucial in regulating lipid and sugar metabolism, but its expression decreases with age in oocytes [239, 240]. Additionally, the down-regulation of miR-21, miR-15a, miR-16, and miR-128 in aging oocytes after ovulation in mice may indirectly affect cellular energy metabolism [241]. miR-21 can affect the PI3K/AKT pathway [242], miR-15a can regulate lipid metabolism by affecting acetyl-CoA [243], miR-16 can regulate the expression of TCA cycle-related proteins in muscle cells [244], and miR-128 can regulate the FoxO3-related pathway in non-alcoholic fatty liver and mitochondrial fusion and fission [245, 246]. However, the impact of these miRNAs on oocyte metabolism has not been validated. Further exploration is needed to understand the changes of ncRNAs in aging oocytes and their effects on energy metabolism.

Over 170 distinct chemical modifications on various types of RNA, collectively known as the RNA epitranscriptome, regulate cellular metabolism through multiple pathways [247]. These RNA modifications are crucial in oocyte maturation and in delaying reproductive aging, as demonstrated by previous studies [207–213]. However, the impact of RNA modifications on energy metabolism within the context of oocyte aging remains unreported.

### Others

The quality of oocytes may be influenced by follicles and their surrounding environment [248]. In vivo, follicles are primarily surrounded by follicular stromal cells, immune cells, nerves, blood vessels, and extracellular matrix [249]. These components can impact follicles by providing biological scaffolds, nutrients, and signaling molecules [249]). In aging ovaries, fibrosis increases, angiogenesis decreases, and an inflammatory microenvironment disrupts the stability of normal ovarian structure [250, 251]. Additionally, aging ovaries experience age-dependent changes in the status of granulosa cells and the composition of follicular fluid [250]. Environmental changes due to aging may affect the energy metabolism of oocytes, such as inducing hypoxic environments in the microenvironment of aging ovaries [252]. Hypoxia can influence cellular metabolic reprogramming through hypoxia-inducible factors (HIFs) [253, 254]. However, hypoxic culture alone cannot replicate the in vivo hypoxic environment of follicles, and the impact of hypoxia on oocyte metabolic reprogramming has not been investigated [254]. Reduced antioxidant levels in follicular fluid and age-related declines in granulosa cell defense against oxidative stress may also result in impaired redox status in oocytes [252, 255–257]. Impaired redox status can also disrupt ATP production through various pathways, impacting oocyte quality [258–260]. As mentioned earlier, oocytes can derive energy substrates from follicular fluid and granulosa cells. Therefore, age-related metabolic changes in granulosa cells and alterations in related nutrients in follicular fluid may influence their supportive role in oocyte nutrition. With aging, there is an upregulation of genes involved in glycolysis, fatty acid metabolism, and cAMP conversion in cumulus cells, while protein expression related to oxidative phosphorylation is downregulated. Normal mitochondria decrease, glucose levels in follicular fluid decrease, lactate levels increase, and apolipoprotein levels and major lipid levels increase [252, 261]. This indicates an increased capacity of cumulus cells to produce substances such as pyruvate, lactate, and AMP, thereby supporting the energy metabolism requirements of oocytes. However, the transport of these substances from cumulus cells to oocytes through the cumulus-oocyte complex decreases with maternal aging [22], potentially reducing the ability of cumulus cells to support oocytes. Therefore, further quantitative studies are necessary to investigate the changes in the nutritional support role of cumulus cells for oocytes. Granulosa cells, in addition to influencing oxidative stress and nutritional support, can also impact specific energy metabolism intermediates. For instance, citrate is a crucial substance in the TCA cycle. The age-dependent decrease in autophagy levels in granulosa cells may affect oocyte quality by reducing follicular fluid citrate levels [37]. However, it is unclear whether this affects the energy metabolism process of oocytes. Exosomes in the follicular fluid can influence oocytes, but their age-dependent changes and their potential regulation of oocyte metabolism lack research. In the natural cycle, there are no significant hormonal differences in follicular somatic cells among elderly women [252]. However, some studies have indicated that with increasing maternal age, the level of follicle-stimulating hormone in follicles increases, potentially impacting the redox activity of oocytes and disrupting oocyte homeostasis [262].

In elderly women, oocytes exhibit characteristics of mitochondrial decline, including dysfunction, reduced quantity, impaired dynamics, accumulation of DNA mutations, decreased ATP production, and increased oxidative stress [263–268]. Furthermore, protein mechanisms controlling mitochondrial health, such as mitochondrial autophagy, are also impaired in the oocytes of elderly women [269, 270]. Mitochondria serve as the central site of cellular energy metabolism, participating in processes such as the TCA cycle, oxidative phosphorylation, and the synthesis of energy metabolism-related proteins [271, 272]. The decline in mitochondrial quality significantly impacts energy metabolism [271, 272].

DNA damage, telomere shortening, and aneuploidy are also signs of oocyte aging [273, 274]. Energy metabolism disorders can impact oocyte aging by affecting oxidative stress [275, 276]. However, the role of energy metabolism in oocytes remains underexplored. Current research primarily investigates the impact of mtDNA damage and repair mechanisms on oocyte energy metabolism. MtDNA damage can disrupt cellular energy metabolism [277]. RNA sequencing analysis suggests that mtDNA expression in oocytes declines with age. Subunits of respiratory chain complex I encoded by mtDNA, such as mt-Nd2, mt-Nd3, mt-Nd4, mt-Nd4L, and mt-Nd5, are downregulated in oocytes of aged mice [40]. Additionally, genes encoding respiratory chain subcomplexes IV and V, ATP synthase 6 (MT-ATP6), cytochrome c oxidase subunit II (COX2), and cytochrome c oxidase subunit III (COX3) in the mitochondrial genome are downregulated in GV oocytes of aged mice [40]. Mitochondrial ribosomes, responsible for translation within mitochondria, exhibit age-dependent downregulation of mitochondrial ribosomal protein L32 (MRPL32) and mitochondrial ribosomal protein L15 (MRPL15) in oocytes [23]. Age-related reduction in mitochondrial DNA levels leads to electron transport chain (ETC) uncoupling, decreased energy supply, and can worsen oxidative stress and inflammation, contributing to aging and disease [278, 279]. While mtDNA expression declines with age in oocytes, it does not necessarily signify mtDNA damage. Experimental validation is necessary to confirm age-dependent mtDNA damage in oocytes. In summary, the precise landscape of age-dependent DNA damage, telomere shortening, and aneuploidy in oocytes remains unclear and warrants further research. Likewise, the relationship between these factors and energy metabolism requires a deeper understanding.

## Treatment

Although there are currently no clinically feasible techniques to maintain or reverse age-related ovarian dysfunction, in recent years, many therapies have shown potential for improving female fertility. Some of these therapies can enhance the quality of oocytes and delay reproductive aging by influencing the energy metabolism of oocytes.

### Antioxidant

Oxidative damage is a key factor in the decline of ovarian function with age, and many treatment strategies aim to counteract ovarian aging through antioxidant interventions. When the levels of ROS in cells exceed their defense capacity, it may affect metabolism through oxidative mtDNA damage and the activation of hypoxia-inducible factor-1α (HIF-1α), among other pathways [280–282]. Therefore, the antioxidant effects of antioxidants may partly improve oocyte energy metabolism and restore oocyte quality. For oocytes, CoQ10 [283–286], polyamines [287–291], melatonin [22, 292–296], ginsenoside Rh2 [297], β-nicotinamide mononucleotide (NMN) [298, 299], resveratrol [300], ferulic acid [301], L-carnitine [163], menaquinone [302], leonine [303], and others can directly act as antioxidants. They stimulate the synthesis of other endogenous antioxidants and influence protein expression to improve mitochondrial function, alleviate age-related oxidative stress damage to oocytes, and enhance oocyte quality. Dietary regulation can also impact oocyte oxidative stress. For example, intermittent fasting can improve oocyte quality by restoring the NAD + /Sirt1-mediated antioxidant defense system, which can eliminateexcessive ROS accumulation [304].

In addition to antioxidation, antioxidants can also improve the quality of aging oocytes by affecting metabolism through other pathways. For example, supplementing with CoQ10 can enhance the mitochondrial function of aging oocytes, restore the activity of the TCA cycle and electron transfer, promote the activation of important energy metabolism proteins such as mitogen-activated protein kinase 14 (MAPK14) and peroxisome proliferator-activated receptor alpha (PPARA), and normalize energy production [11, 286, 305]. Polyamines, such as spermidine, putrescine, and spermine, are a group of poly-cationic aliphatic amines that are widely found in mammals. They interact with negatively charged biomacromolecules and play a crucial role in ovarian aging. [290, 291, 306–308]. In addition to antioxidation, polyamines can regulate oocyte energy metabolism through various pathways. For example, spermidine can increase mitochondrial respiration, promote translation of respiratory chain complex IV subunit cytochrome c oxidase subunit 4 (COX4), activate mitochondrial respiratory chain complexes I, III, and IV, induce upregulation of key energy metabolism protein Hif-1α and activation of AMPK [289, 307–312]. Eukaryotic translation initiation factor 5A (eIF5A) is a highly conserved protein that contributes to the expression of various mitochondrial proteins, and some of these proteins are involved in the TCA cycle and OXPHOS. Its deficiency can cause reduced oxygen consumption, ATP production, levels of mitochondrial metabolic enzymes, and altered mitochondrial dynamics [313, 314]. Spermidine is a substrate for eIF5A hydroxylation, and polyamines may partially exert their effects on mitochondria through eIF5A [315, 316]. Spermine can restore depolarized mitochondria membrane potential and inhibit lipid formation [317, 318]. Putrescine can also improve fatty acid oxidation, regulate glucose metabolism, and enhance mitochondrial activity [290, 291]. The increased circulation of polyamines is related to increased glucose and lipid metabolism [319]. On the other hand, spermidine/spermine N1-acetyltransferase (SAT1) is the downregulation of the rate-limiting enzyme of polyamine catabolism, which can increase the availability of acetyl-CoA in fatty acid synthesis by reducing acetyl-CoA consumption during polyamine acetylation, leading to decreased oxidative phosphorylation [320, 321]. The activation of SAT1 expression is associated with lipid synthesis, triggers lipid peroxidation, and enhances cellular susceptibility to ROS-induced stress. [322]. Additionally, depletion of polyamines in cells leads to glycolysis inhibition, suppression of the TCA cycle and OXPHOS, decreased levels of acetyl-CoA and histone acetylation, downregulation of mitochondrial metabolism genes, downregulation of TCA intermediates and OXPHOS activity, upregulation of stress signaling, and enhanced mitochondrial permeability [323, 324]. For oocytes, the level of putrescine decreases with age and is one of the main factors for poor quality in the oocytes of aged mouse [325]. Supplementing with putrescine can enhance the quality of aging mouse oocytes, reduce aneuploidy, improve embryo quality, and decrease the miscarriage rate in aged mice [326–329]. Spermidine restores oocyte quality in female reproductive aging by enhancing mitochondrial autophagy [330]. Therefore, supplementing with polyamines can regulate the age-related decline in oocyte quality, partly by affecting oocyte energy metabolism. It is worth noting that in mouse experiments, supraphysiological doses of spermidine induce ovarian oxidative stress and granulosa cell apoptosis [331]. The impact of supplementing with spermidine on oocyte quality has not been fully studied. Further animal and clinical studies are required to understand the effects of supplementing with polyamines on age-related changes in oocyte quality. Additionally, NMN supplementation can restore NAD + levels, thereby enhancing ovulation and fertilization capacity [332].

### Mitochondrial therapy

Mitochondrial therapy has been considered a potential treatment option for improving oocyte quality in infertile patients. The decline in the expression of important proteins and RNA encoded by mtDNA in oocytes, along with the increase in mutations, may lead to decreased fertility with age, as well as harmful effects on offspring, possibly through the impact on mitochondrial function and susceptibility to oxidative damage [65]. Supplementing the copy number of mtDNA can improve oocyte quality [66–68]. Mitochondrial genome editing (MGE) through mtDNA editing holds great potential for treating mtDNA-related diseases [333]. Mitochondrial replacement therapy is also capable of reducing the accumulation of mitochondrial DNA abnormalities, improving mitochondrial function, and keeping the high ATP demand required by oocytes, thereby reversing the decline in oocyte quality, although its methods are quite invasive [334–338].

### Others

In recent years, many therapies have been proven to improve the processes related to oocyte energy metabolism. For example, brown adipose tissue-derived exosomes can prevent the deterioration of mitochondrial function caused by oocyte aging in mice and increase the ATP content in oocytes [339]. Salidroside can promote lipid metabolism, improve mitochondrial function, and enhance the maturation of pig oocytes [340]. Dehydroepiandrosterone activates energy metabolism in the theca and granulosa cells, thereby transferring energy to oocytes and promoting oocyte regeneration [341]. Growth hormone has a repairing effect on mitochondrial function in oocytes and can directly or indirectly promote oxidative stress balance and cellular antioxidant defense, as well as facilitate oxidative phosphorylation pathways [342]. FoxO3a is an important factor in regulating oocyte metabolism, and curcumin can regulate the PTEN/AKT/FoxO3a pathway to protect ovarian reserve [198]. However, further clinical and basic experiments are needed to determine the relevant mechanisms and clinical effects of these therapies.

## Conclusions

The decline in fertility associated with women’s increasing age has become a significant issue for modern women [1–4]. As age increases, there is a decrease in ATP within the oocytes, leading to a decline in energy production capacity and mitochondrial function [8–15]. The changes in energy metabolism of oocytes with age have an impact on oocyte quality and are an important mechanism of reproductive aging [16–18]. However, it is still unclear how the changes in the intermediate steps of energy metabolism in oocytes affect ATP generation and how the related products and proteins influence oocyte quality, thus affecting ovarian aging. Therefore, this review summarizes the characteristics of oocyte energy metabolism, the changes in the TCA cycle, oxidative phosphorylation, lipid metabolism, glutamine metabolism, and the Adenosine remedial pathway in oocytes during age-related ovarian aging, as well as how these changes affect oocyte quality. This review also introduces the important proteins SIRTs and FoxO3a that regulate oocyte metabolism. Finally, this review discusses some treatment strategies for delaying ovarian aging, which may partially act by influencing oocyte energy metabolism. In conclusion, understanding the changes in oocyte metabolism and their influence on oocyte quality in age-related ovarian aging helps us comprehend the relationship between oocyte quality decline and the subsequent decline in fertility, and aids in identifying biomarkers and treatment methods.

## Data Availability

The datasets used and/or analyzed during the current study are available in the MEDLINE repository: https://pubmed.ncbi.nlm.nih.gov/. All figures were depicted by PowerPoint.

## Abbreviations

ACAA: 3-Ketoacyl-CoA thiolase
Acytl-CoA: Acetyl coenzyme A
ADP: Adenosine diphosphate
AKT: Ak Strain Transforming
AMP: Adenosine monophosphate
AMPK: AMP-dependent protein kinase
ANT1: ADP/ATP translocase 1
ATP: Adenosine 5'-triphosphate
CACT: Carnitine–acylcarnitine–translocase
CD36: Cluster of Differentiation 36
COX4: Cytochrome c oxidase subunit 4
CpG: Cytosine-phosphate-guanine
CPT1: Carnitine Palmitoyltransferase 1
CPT2: Carnitine Palmitoyltransferase 1
COX: Cytochrome c oxidase subunit II
CoQ: Coenzyme Q
DAG: Diacylglycerol
DGAT1: Diacylglycerol o-acyltransferase 1
DNA: Deoxyribonucleic acid
DNMT: DNA methyltransferases
DOR: Diminished ovarian reserve
EHHADH: Enoyl-CoA hydratase 3-hydroxy acyl-CoA dehydrogenase
eIF5A: Eukaryotic translation initiation factor 5A
ETC: Electron transport chain
FA: Fatty acid
FABP3: Fatty acid-binding protein 3
FADH2: Flavin adenine dinucleotide
FAO: Fatty acid β-oxidation
FATP: Fatty acid transport protein
FA-CoA: Fatty acyl-CoA
FOXO3a: Forkhead box O3a
GDH: Glutamate dehydrogenase
GH: Growth hormone
GLS: Glutaminase
GS: Glutamine synthetase
HDAC: Histone Deacetylase
HIF-1α: Hypoxia-inducible factor-1α
HOXD8: Homeobox D8
IGF: Insulin-like Growth Factor
KAT8: Lysine acetyltransferase 8
LCFA-CoA: Long-chain acyl-CoAs
LysoPL: Lysophospholipid
MAPKAP1: Mitogen-activated protein Kinase Associated Protein 1
MAPK14: Mitogen-activated protein kinase 14
MGE: Mitochondrial genome editing
MRPL: Mitochondrial ribosomal protein L
Mito: Mitochondria
mtDNA: Mitochondrial DNA
mTOR: Mammalian Target of rapamycin
NADH: Nicotinamide adenine dinucleotide
NADPH: Nicotinamide adenine dinucleotide phosphate
ncRNA: Non-coding RNA
NMN: β-Nicotinamide mononucleotide
NR: Nicotinamide riboside
NR1D1: Nuclear receptor subfamily 1, group D, member 1
OXPHOS: Oxidative phosphorylation
PARP: Poly(ADP-ribose) polymerase
PDE: Phosphodiesterase
PI3K: Phosphoinositide 3-kinase
PL: Phospholipid
PMF: Proton-motive force
PPP: Pentose phosphate pathway
PPARA: Peroxisome proliferator-activated receptor alpha
rDNA: Ribosomal DNA
ROS: Reactive oxygen species
SAT1: Spermidine/spermine N1-acetyltransferase
SIRTs: Sirtuin enzyme family
SLC14A1: Solute carrier family 14 member 1
TAGs: Triacylglycerols
TCA cycle: Tricarboxylic acid cycle
Tcf20: Transcription co-activator factor 20
5-mC: 5-Methylcytosine
